# A Study on the Mechanism and Properties of a Self-Powered H_2_O_2_ Electrochemical Sensor Based on a Fuel Cell Configuration with FePc and Graphene Cathode Catalyst Materials

**DOI:** 10.3390/bios14060290

**Published:** 2024-06-04

**Authors:** Yunong Zhang, Andreas Offenhäusser, Yulia Mourzina

**Affiliations:** 1 Institute of Biological Information Processing—Bioelectronics (IBI-3), Forschungszentrum Jülich, 52425 Jülich, Germany; yun.zhang@fz-juelich.de (Y.Z.); a.offenhaeusser@fz-juelich.de (A.O.); 2 RWTH Aachen University, 52062 Aachen, Germany

**Keywords:** self-powered electrochemical sensor, H_2_O_2_ sensor, fuel cell, biomimetics, metal phthalocyanine, graphene, serum analysis, standard addition method

## Abstract

Conventional electrochemical sensors use voltammetric and amperometric methods with external power supply and modulation systems, which hinder the flexibility and application of the sensors. To avoid the use of an external power system and to minimize the number of electrochemical cell components, a self-powered electrochemical sensor (SPES) for hydrogen peroxide was investigated here. Iron phthalocyanine, an enzyme mimetic material, and Ni were used as a cathode catalyst and an anode material, respectively. The properties of the iron phthalocyanine catalyst modified by graphene nanoplatelets (GNPs) were investigated. Open circuit potential tests demonstrated the feasibility of this system. The GNP-modulated interface helped to solve the problems of aggregation and poor conductivity of iron phthalocyanine and allowed for the achievement of the best analytical characteristics of the self-powered H_2_O_2_ sensor with a low detection limit of 0.6 µM and significantly higher sensitivity of 0.198 A/(M·cm^2^) due to the enhanced electrochemical properties. The SPES demonstrated the best performance at pH 3.0 compared to pH 7.4 and 12.0. The sensor characteristics under the control of external variable load resistances are discussed and the cell showed the highest power density of 65.9 μW/cm^2^ with a 20 kOhm resistor. The practical applicability of this method was verified by the determination of H_2_O_2_ in blood serum.

## 1. Introduction

Hydrogen peroxide (H_2_O_2_) is not only widely used in the medical, health, industrial, and domestic fields, but it also plays an important role in the regulation of biological activities [1,2,3,4]. Moreover, H_2_O_2_ is a product of oxidoreductase-catalyzed transformations; its quantification is used for the detection of other biologically important markers, e.g., glucose, lactate, uric acid, and glutamate [5,6,7]. Therefore, the detection of H_2_O_2_ has been widely studied with different methods, such as fluorescence, spectrophotometry, and electrochemistry [8,9,10]. Of all the aforementioned methods, the electrochemical detection is rapidly advancing due to its advantages in terms of simplicity, sensitivity, selectivity, response rate, portability, and wearable sensor technology.

Conventional electrochemical sensors usually consist of different parts that undertake different functions. The most important units are commonly the electrochemical cell and the external power supply system. The electrochemical cell contains a working electrode, a counter electrode, and a reference electrode, which assumes the roles of identification and signal generation. Since the signal of the analyte is generally obtained by voltammetric or amperometric methods, an external power supply system is essential, as it provides a polarization energy to apply and control the external potential on the electrodes to drive a particular redox indicator reaction [11,12]. In this way, the electrochemical sensor requires an external power and modulation system, which makes it relatively complex, expensive, and unsuitable for flexible design and practical applications [13]. Self-powered electrochemical sensors (SPESs) are therefore attracting increasing attention [12,13,14,15,16].

A fuel cell-based SPES is a galvanic electrochemical system that generates a signal by using analytes as fuel and converts chemical energy directly into electrical energy through thermodynamically favorable electrochemical reactions, which makes the external power supply redundant. Furthermore, as the signal is not obtained by voltammetric or amperometric methods, the reference electrode in the electrochemical cell is not required, thus making the electrochemical cell simpler. The whole sensor system therefore contains fewer units, saves energy, and is more compatible for applications in wearable equipment as well as in in vivo and in situ tests. Despite its advantages, it is worth noting that a closed galvanic circuit is crucial for a functional fuel cell. As a result, two reactions including an oxidation reaction on an anode and a reduction reaction on a cathode are required, and a suitable electrolyte is also necessary for internal electron transfer. The performance of fuel cell-based self-powered electrochemical sensors is thus strongly reliant on the condition of the medium, the electrochemical properties of the cathode and anode materials, and the structure of the electrodes. Moreover, ion-exchange membranes are often used in fuel cells to separate anodic and cathodic electrolytes. However, for sensor applications, systems without a membrane are more attractive.

To develop fuel cell-based, self-powered H_2_O_2_ sensors, it is essential to find suitable fuel cell systems that can selectively and efficiently consume H_2_O_2_ as fuel to generate electrons and to realize the output of the signal. In the case of galvanic cells, polarization overvoltages for the electrode reactions make a negative contribution to the electrical energy output and can lead to a lack of sensing properties at a negative cell potential in the case of high overvoltages, reducing the cell voltage according to Equation (1) [17,18]:

E_d_ = E_0_ − η_a_ − |η_c_| − I·∑R = E_0_ − η_cell_, (1)

where E_d_—is the cell voltage during current flow (discharge potential); E_0_ = E_c_ − E_a_—is the cell voltage in the absence of current flow in the cell, also known as open circuit voltage; E_c_ and E_a_—are the cathode and anode potentials, respectively; η_c_ and η_a_—represent the polarization of the cathode and anode, respectively; I—is the current in the operating mode of the cell; and ∑R—is the sum of the internal and external resistances except for the polarization resistances taken into account by η.

The electrodes are therefore modified with the catalyst to improve the thermodynamics and kinetics of the electrode reactions as well as the analytical characteristics of the respective sensors, such as natural enzymes [3,8,19,20], non-enzymatic electrocatalysts including metal nanostructures [21,22,23], carbonaceous nanomaterials, enzyme mimetics, and nanozymes such as metallocomplexes and Prussian blue [6,7,8,24,25,26,27]. Of the aforementioned materials, enzymatic catalysts have advantages in terms of specificity and high catalytic activity, which are determined by the properties of the enzymes. However, the purification and immobilization processes of the enzymes are problematic, and the operating conditions of the enzymatic electrodes are strictly limited. Moreover, the long-term stability of the enzymatic sensors is also affected by their strict storage conditions and it is difficult to modify the enzymatic materials to tune their properties. In this case, non-enzymatic materials, enzyme mimetics, and nanomaterials become an attractive option for researchers.

Biomimetic (electro)catalytic systems based on transition metal complexes with macroheterocyclic phthalocyanine (MPc) ligands belong to a class of MN4 catalysts, which have attracted a great deal of attention due to their structural and functional similarities to peroxidase enzymes as well as their (electro)catalytic function in many reactions, in particular in the reduction of oxygen and hydrogen peroxide. The high catalytic activity of these compounds is due to the presence of a heteroaromatic π–system of the macrocycle and its interaction with the central metal atom, the ability of the central metal to change its valence states, the possibility of coordinating extra ligands in axial positions, and the tuning of the environment of the central metal atom using substituents of the macrocycle. However, some properties of phthalocyanines, such as pure conductivity and a tendency to aggregation, may have a negative impact on electrocatalytic activity. To solve this problem, MPcs can be supported on carbonaceous nanomaterials.

Previously, a series of studies reported on the research into H_2_O_2_ fuel cells in acidic solutions with different cathode materials such as metallophthalocyanine and Prussian blue [28,29,30,31,32]. To fully understand the advantages and shortcomings of applying fuel cell-based, self-powered systems in sensor fields, in this paper, we report on a novel fuel cell-based H_2_O_2_ SPES using H_2_O_2_ as a single fuel by employing iron phthalocyanine (FePc) and FePc supported on graphene material as cathode catalysts and Ni as anode material. The morphology and modification of FePc are presented with respect to the improved sensor performance. Since both the cathode catalyst and the anode catalyst had a separate and collective influence on the performance of the sensor, the electrochemical performance of the FePc, graphene-supported FePc, and Ni electrodes are discussed under different conditions. More importantly, the mechanism and detection function of the self-powered sensor are presented and discussed in detail.

## 2. Materials and Methods

### 2.1. Materials and Reagents

Glassy carbon electrodes (GCEs) with a working electrode diameter of 3 mm were purchased from ALS Co., Ltd. (Tokyo, Japan). FePc (>97%) and GNP were purchased from Tokyo Chemical Industry Co., Ltd. (Tokyo, Japan). The pH 7.4 phosphate buffer solutions were prepared from NaH_2_PO_4_ and Na_2_PO_4_. The pH 3 buffer solutions were prepared from H_3_PO_4_ and NaH_2_PO_4_. Pt wire (diameter 1 mm, >99.99%), Ni wire (diameter 0.5 mm, >99.99%), phosphates, potassium chloride, 30% H_2_O_2_ solution, 5% Nafion solution, heat-inactivated human blood serum, DMF solvent, and other chemical reagents were purchased from Merck KGaA (Darmstadt, Germany). Reagents were of analytical grade. H_2_O_2_ solutions were prepared before measurements. All solutions were prepared using deionized water (Milli-Q water purification system; Merck KGaA, Darmstadt, Germany).

### 2.2. Electrode Preparation

GCEs were washed with ethanol and water, polished using 0.1 µm and 0.05 μm polishing powders, then washed with deionized water.

FePc was dissolved in DMF at 0.6 mg/mL, GNP was dispersed in DMF at 3 mg/mL, and the dispersion was ultrasonicated. The mixture of GNP–FePc contained 3 mg/mL GNP and 0.6 mg/mL FePc in DMF. The GNP–FePc mixture was put on a rotator for 3 h to fully mix the materials, and the FePc solution was also treated the same way to reduce errors.

After 3 h, 7 µL of FePc, GNP, or GNP–FePc were dropped on GCEs, and the GCEs were subsequently dried at 60 °C for 40 min. A total of 7 µL of 0.33% Nafion solution (diluted by DMF) was then dropped on the GCEs and dried at 60 °C for 40 min. The modified GCEs were subsequently used after cooling them down to room temperature.

### 2.3. Electrochemical Experiments

All experiments were performed in duplicate to quadruplicate.

Electrochemical experiments were conducted using an Autolab PGSTAT (Metrohm Autolab B.V., Utrecht, The Netherlands) in a three-electrode glass cell with a coiled platinum wire as a counter electrode and an Ag|AgCl|KCl 3 M:0.5 M KCl double-junction reference electrode (Metrohm, Herisau, Switzerland). If not otherwise stated, the scan rate was 50 mV/s. EIS was conducted using a Biologic VSP-300 potentiostat (Biologic, Seyssinet-Paiset, France) and the results were analyzed by EC-Lab software V11.50 (Biologic, France).

For measurements under deoxygenated conditions, the solutions were deaerated by Ar flow for 20 min prior to the experiment and a stream of Ar was passed over the solutions during the experiment. The standard deviation of the potential values in the CV experiments did not exceed 5%.

The CV tests were conducted in 15 mL phosphate buffer solutions at room temperature. If not otherwise stated, the scan rate was 50 mV/s. H_2_O_2_ was added to buffers after CV tests without H_2_O_2_. Ni wire was immersed in 0.1 M H_2_SO_4_ for 15 min before each measurement and washed with DI water.

Electrochemical impedance spectroscopy (EIS) tests were conducted in 15 mL phosphate buffer solutions at room temperature. The solutions were deoxygenated with Ar for 20 min before measurement. The frequency range was 100–2 × 10^6^ Hz, the AC voltage was 0.01 V, and the DC voltages were 0.28 V at pH 3.0 and 0.215 V at pH 7.4, which were determined by the E_1/2_ obtained in the CV tests.

Open circuit potential (OCP) tests. The tests of the OCP of Ni against Ag/AgCl and FePc (GNP–FePc) against Ag/AgCl were conducted in phosphate buffer solutions with a salt bridge. For Ag/AgCl, a 15 mL 0.05 M phosphate buffer with 0.1 M KCl was used. For Ni and FePc (GNP–FePc), a 15 mL 0.1 M phosphate buffer was used and it was mechanically stirred at 300 rpm. H_2_O_2_ was only added to a 0.1 M phosphate buffer of the Ni or FePc (GNP–FePc) half-cells, meaning that the buffer for Ag/AgCl remained almost unchanged during the test.

For the OCP of the SPES cell, Ni and FePc (GNP–FePc) were used as the anode and the cathode in 15 mL 0.1 M phosphate buffer, with 300 rpm mechanical stirring and the additions of H_2_O_2_. The addition of H_2_O_2_ started at 250 s with an interval of 50 s. The signal was recorded by Autolab with an open circuit, meaning that no electron transfer took place from the anode to the cathode.

Self-powered electrochemical sensor property tests. The tests were conducted in a one-compartment cell that used Ni wire as an anode and FePc (GCE–FePc) as a cathode with 0.1 M phosphate buffer. The solution was mechanically stirred at 300 rpm and the addition of H_2_O_2_ started at 250 s with an interval of 50 s. The dependence of the current between the anode and cathode on time was recorded.

For the power output tests, a multimeter was used to record the current and the potential changes. Adjustable resistors were connected to the circuit in series (for current measurements) or in parallel (for potential measurements), and the experiment started with an external resistance of 340 kOhm. A deoxygenated 0.1 M pH 3.0 phosphate buffer solution with 3 mM H_2_O_2_ was used and stirred during the test. GNP–FePc and Ni wire were used as a cathode and anode, respectively. 

For the sensor ability tests with different external variable load resistors, the process was similar to the tests without resistors, but a resistor with fixed resistance was connected.

For the interference tests, the process was similar to the tests described above, the tests were performed with the GNP–FePc cathode, and the addition of different chemicals started at 250 s with an interval of 100 s.

Detection of hydrogen peroxide in blood serum. In the experiments on the determination of hydrogen peroxide in serum samples, the standard addition method [33] and apparent recovery [34] were used. The serum was diluted 1:2 *v*/*v* with 0.1 M phosphate (pH 3) buffer. Known amounts of hydrogen peroxide were added to the diluted serum samples and the concentration of hydrogen peroxide was determined using a four-point standard addition method. The dependence of the current between the anode and cathode in the SPES configuration was recorded as a signal. The values of the current recorded in the solution with the “unknown” concentration of hydrogen peroxide and in the solutions with four additions of the known concentration of hydrogen peroxide were plotted against the hydrogen peroxide concentration. A graph was used to determine the “unknown” concentration of hydrogen peroxide and the apparent recovery. The measurements were repeated three times for each “unknown” concentration of hydrogen peroxide.

Further experimental details can be found in the Supplementary Materials.

## 3. Results and Discussion

### 3.1. Mechanism

The decomposition of H_2_O_2_ is given by Equation (2):

2H_2_O_2_ → 2H_2_O + O_2_ (2)

To generate electricity, the reactions on the anode and the cathode of a fuel cell are the following [35]:

Anode: H_2_O_2_ → 2H^+^ + O_2_ + 2e^−^      E^0^_a_ = 0.68 V (3)

Cathode: 2H^+^ + H_2_O_2_ + 2e^−^ → 2H_2_O    E^0^_c_ = 1.77 V (4)

Therefore, when the oxidation potential of H_2_O_2_ at the anode is relatively negative and the reduction potential of H_2_O_2_ on the cathode is relatively positive, then E_c_ − E_a_ > 0. This corresponds to a negative change in the Gibbs free energy of the process and allows the H_2_O_2_ fuel cell to work. The current output can then be used as an identification signal to detect the concentration of H_2_O_2_.

### 3.2. Morphology Analysis

**Figure 1:**
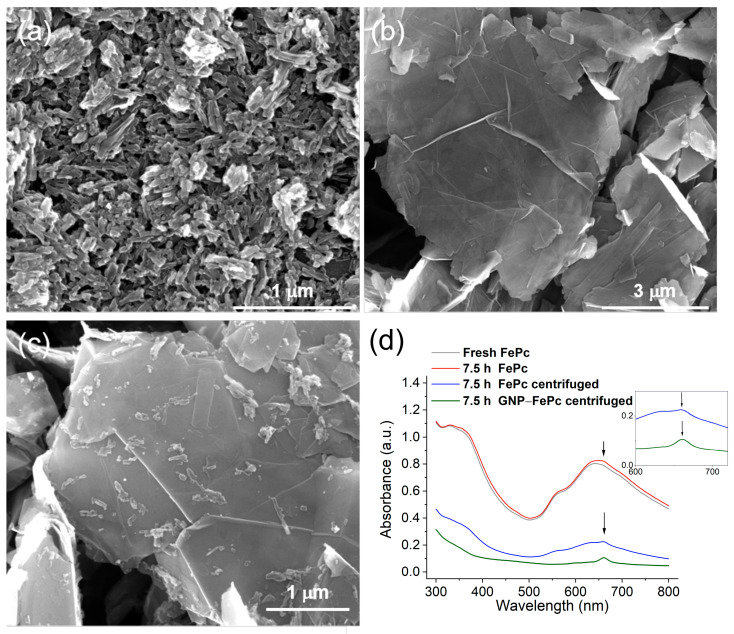
SEM images of (**a**) FePc, (**b**) GNP, (**c**) GNP–FePc, and (**d**) UV–Vis spectra of different samples; the insert shows the enlarged areas of the spectra and arrows mark that a new peak appeared at about 660 nm.

The component ratio of the GNP–FePc composite was optimized in the preliminary experiments (Figure S1a). The structure and surface morphology of FePc and GNP–FePc were analyzed by means of SEM (Figure 1a–c). The stability and interaction between FePc and GNP were monitored by UV–Vis (Figure 1d). 

The SEM test revealed that FePc formed rod-like structures with a diameter of 30–40 nm on a glassy carbon (GC) substrate (Figure 1a). Although the FePc was well distributed on the surface of the GC substrate, it should be noted that the FePc was aggregated, which was caused by the dimerization effect and the self-assembly of FePc at a high concentration in a DMF solvent [36]. The aggregation of FePc could have resulted in an inferior electrochemical activity, as the active iron centers were enclosed by phthalocyanine rings and less exposed due to aggregation. 

Figure 1b displays a sheet-like structure with separated layers and “wrinkles” of the GNP on the GC substrate. Compared to the rod-like structure of FePc, GNP is larger, which enables GNP to adsorb FePc on its surface due to π-π interactions. The adsorption of FePc on GNP hinders intermolecular interaction and the aggregation of FePc molecules, and it results in an essentially better dispersion and a better exposure of catalytic Fe centers. It can be seen in Figure 1c that after mixing GNP and FePc in DMF for several hours, FePc was attached to the GNP surface with less aggregation. This significant improvement in the dispersion of the FePc catalyst could have been caused by the interaction between conjugated π-electron systems of the graphene and phthalocyanine rings.

Figure 1d shows the electronic spectra of the FePc and GNP–FePc samples in the UV–Vis wavelength region. For the fresh FePc solution, a peak at around 640 nm indicates the structure of ferrous phthalocyanine. After 7.5 h, a new peak appeared at about 660 nm (marked by arrows), indicating a structure of ferric phthalocyanine and the oxidation of Fe(II)Pc by O_2_ in DMF [36,37,38]. The blue and green lines in Figure 1d were obtained by detecting the spectra of the liquid after centrifugation. It shows that the supernatant of the centrifuged FePc had a stronger absorption intensity than the supernatant of the centrifuged GNP–FePc, which indicates that FePc was absorbed and then precipitated with GNP.

Figure S1b–f show the EDX spectra and elemental mapping of the GC, GNP, and GNP–Fe composite.

### 3.3. Electrochemical Studies of the Cathode

Cyclic voltammetry (CV) tests were carried out under different conditions to investigate the electrochemical performances of different cathodes. The tests were first conducted in deoxygenated pH 3.0 and pH 7.4 buffers, as pH 3.0 is the acidic medium generally used in H_2_O_2_ fuel cells, while for sensor applications, a pH 7.4 medium is also of interest.

**Figure 2:**
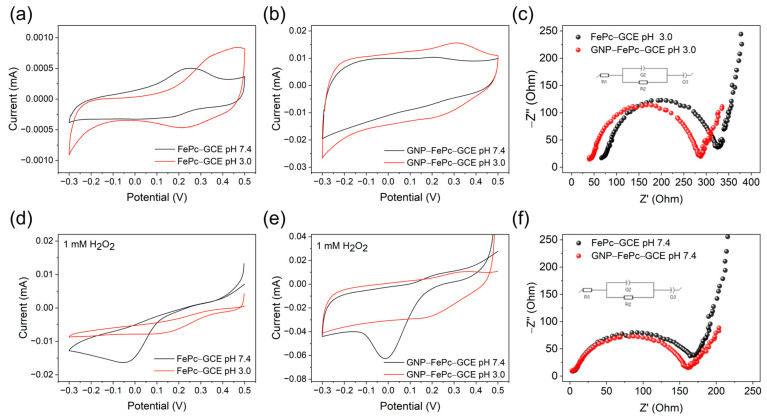
Voltammetric and EIS characterization of cathodes in deoxygenated 0.1 M phosphate buffers. Representative cyclic voltammograms of (**a**) FePc, (**b**) GNP–FePc, (**d**) FePc with 1 mM H_2_O_2_, and (**e**) GNP–FePc with 1 mM H_2_O_2_. The scan rate was 50 mV/s. (**c**,**f**) EIS at pH 3.0 and pH 7.4.

The CVs of FePc and GNP–FePc on GCE in the deoxygenated solutions in Figure 2a,b indicate a quasi-reversible redox process of Fe(III/II)Pc. The electrochemical properties of the aforementioned electrodes are summarized in Table 1. These results show a direct electron transfer between GCE and Fe(III/II)Pc or a GNP–Fe(III/II)Pc coating layer, which is an advantage of the biomimetic FePc material over natural enzyme catalysts in biosensors and biofuel cells. This direct electron transfer is generally impeded in enzymatic catalytic materials due to the insulating macromolecular shells of enzymes, and an extra mediator is required.

Figure 2a,b show that the oxidation and reduction peaks of FePc and GNP–FePc shift to a more positive range at pH 3.0 compared with the results at pH 7.4. The ΔE values (Table 1) are larger under acidic conditions, which indicates that protonation influences the reduction process of Fe active centers [39,40]. With the modification of GNP, ΔE decreased and −I_pc_/I_pa_ increased at both pHs (Table 1). This improvement indicates a better reversibility of the Fe(III/II)Pc redox process on the GNP–FePc interface and is explained by the reduced aggregation of FePc, higher exposure, and better contact of the Fe active sites of FePc with the conductive GNP interface. 

The results of the dependence of CV on the scan rate (Figure S2) prove the surface-bound behavior according to a linear dependence of the I–scan rate.

To consume H_2_O_2_ as a fuel, the reduction potential of H_2_O_2_ on a cathode needs to be relatively positive. CV tests were conducted with 1 mM H_2_O_2_ in different deoxygenated solutions. The results are shown in Figure 2d,e. It can be seen that, at both pH, the reduction of H_2_O_2_ on GNP–FePc starts at relatively positive potentials, which indicates that the GNP–FePc complex is beneficial to the reduction process of H_2_O_2_. However, it should be noted that the reduction of H_2_O_2_ on FePc and GNP–FePc was more pronounced at pH 7.4. Nevertheless, the reduction process starts in the more positive range at pH 3.0, which is thermodynamically advantageous for fuel cell construction. We assume that this behavior comes from a collective influence including a stronger oxidation ability of H_2_O_2_ and the protonation condition of phthalocyanine rings at pH 3.0.

As can be seen in Figure S3, there is no pronounced H_2_O_2_ reduction peak at a bare GCE and GNP–GCE, which demonstrates that the reduction currents for the electrodes modified with FePc were caused by the electrocatalytic biomimetic function of FePc. The reduction of H_2_O_2_ at modified electrodes started after the reduced forms of Fe(II)Pc were electrochemically generated at the electrode and proceeded via the electrocatalytic redox cycle, as can be schematically presented in the following reaction sequence [6,41]:

Fe(III)Pc + e^−^ → Fe(II)Pc (5a)

Fe(II)Pc + H_2_O_2_ → (H_2_O_2_)Fe(II)Pc (5b)

(H_2_O_2_)Fe(II)Pc → O = Fe(IV)Pc/O = Fe(III)Pc ^•+^ + H_2_O (5c)

O = Fe(IV)Pc/O = Fe(III)Pc ^•+^ + e^−^ + H^+^ → (OH)Fe(III)Pc (5d)

Fe(II)Pc + 1/2H_2_O_2_ → (OH)Fe(III)Pc (5e)

(OH)Fe(III)Pc + H^+^ → Fe(III)Pc + H_2_O (5f)

where the final Fe(III)Pc from (5f) could again be reduced on the electrode according to (5a).

The results of the EIS experiments are displayed in Figure 2c,f, and the fitting details can be found in Figure S4 and Table S1. The results show that the charge transfer resistance on the electrode is smaller for GNP–FePc than the cases with FePc, indicating a better conductivity with GNP modification and reversibility of the Fe(III/II)Pc reaction, as was also found in the CV experiments.

To gain a better understanding of the functioning mechanism and the properties of the cathode, CV tests were also performed under aerobic conditions. The results are discussed in Section S2 and Figure S5. In summary, the voltammetric studies of cathodic materials under both aerobic and anaerobic conditions illustrate that the current response was enhanced with the GNP modification and oxygen could have had an influence on the cathode.

The detailed discussion of the electrochemical properties of the Ni anode can be found in Section S3 and Figures S6 and S7.

### 3.4. Open Circuit Potential (OCP) Tests of H_2_O_2_ SPES

To further study the mechanism and prove the feasibility of H_2_O_2_ SPES based on the FePc and GNP–FePc electrocatalysts as cathode materials and Ni as an anode, the OCPs of the cathode and anode half-cells were measured separately against the Ag/AgCl reference electrode (RE) as E_c_ (RE/electrolyte with salt bridge/FePc–GCE), E_c_ (RE/electrolyte with salt bridge/GNP–FePc–GCE), and E_a_ (RE/electrolyte with salt bridge/Ni). The OCPs of the cathodes and anode were measured in a two-compartment cell using a salt bridge to avoid the influence of H_2_O_2_ on the Ag/AgCl reference electrode. Furthermore, the OCPs of the H_2_O_2_ SPES were also measured in a one-cell configuration.

**Figure 3:**
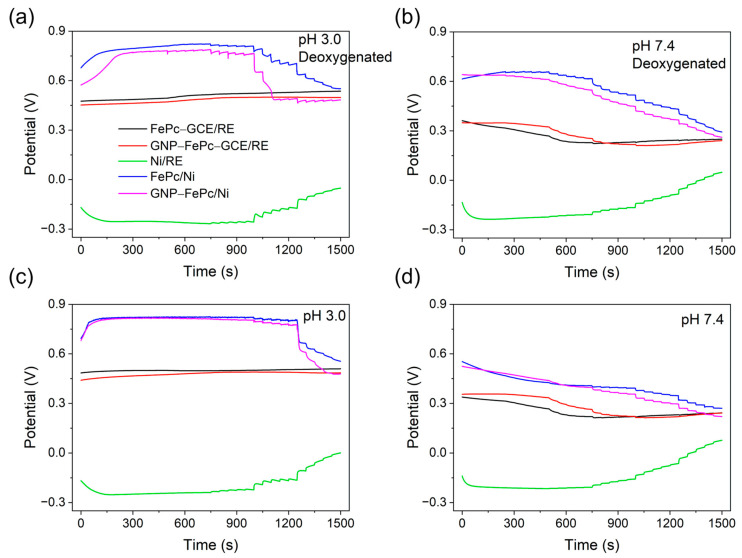
Representative OCP test results of the cathodes or Ni anode (green lines) against Ag/AgCl (RE) and the FePc or GNP–FePc cathodes against the Ni anode. The additions of H_2_O_2_ began at 250 s (C_H2O2_ = 2.2 × 10^−7^ M) and continued every 50 s to C_H2O2_ = 1.1 × 10^−2^ M: (**a**,**b**) deoxygenated conditions and (**c**,**d**) air-saturated buffer.

Figure 3 demonstrates that the E_c_ measured as the OCPs of electrodes with FePc-based catalysts (black and red lines) and H_2_O_2_ against the Ag/AgCl electrode slightly increased at pH 3.0, while it decreased with an increase in the H_2_O_2_ concentration at the beginning and then increased slightly at pH 7.4, thus indicating changes in the dynamics. It should be emphasized that in this open circuit configuration, FePc cannot be reduced and it becomes oxidized by H_2_O_2_, i.e., the cathodes are not inert. It was therefore not possible to understand the mechanism of OCP changes through the Nernst equation. Nevertheless, the change in the oxidizing power of the cathode half-cell (with respect to the anode of the SPES) was likely due to the oxidative degradation of the FePc catalyst and Nafion with H_2_O_2_ [42,43]. In contrast, E_a_ measured as the OCP of the Ni anode (green line, against RE) became more positive with an increase in the H_2_O_2_ concentration, illustrating that the reducing power of the anode half-cell (with respect to the cathode of the SPES) decreased with an increase in the H_2_O_2_ concentration.

The blue and pink lines in Figure 3 show the experimentally measured OCP values of the one-compartment cells composed of the corresponding cathode and anode GCE/FePc/electrolyte + H_2_O_2_/Ni and GCE/GNP–FePc/electrolyte + H_2_O_2_/Ni, respectively. With the collective influence from the OCP changes in the cathode and the anode, a decrease in the OCP of the cell was recorded with an increased concentration of H_2_O_2_. Nevertheless, all curves confirm the positive OCP values (E_c_–E_a_) of the galvanic circuits. The mechanism of the SPES can thus be explained by the electrocatalytic reduction of hydrogen peroxide according to the reactions (5a)–(5f). Meanwhile, the oxidation of hydrogen peroxide and the product of its decomposition, superoxide radical anion (reaction (S12)), took place at the Ni anode at more negative potentials (discussed in Section S3), supplying electrons to the galvanic cell circuit. Moreover, this experiment demonstrates the dependence of the OCP of a cell on the concentration of H_2_O_2_.

### 3.5. Sensor Capability Tests

**Figure 4:**
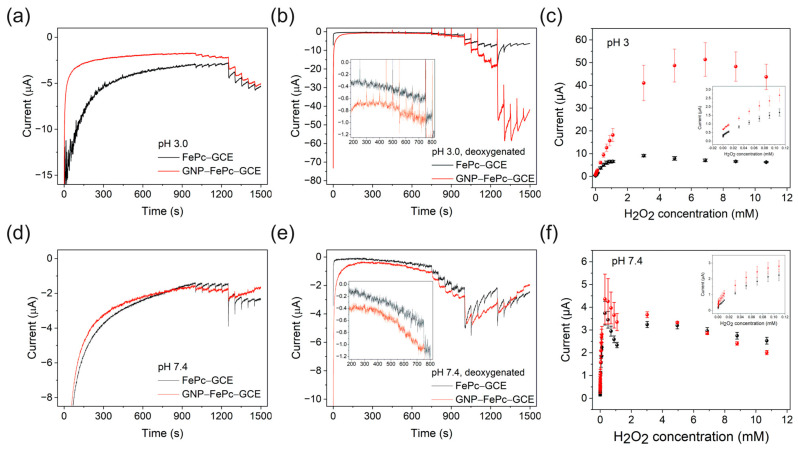
(**a**,**b**,**d**,**e**) Representative signal responses of the self-powered configuration in 0.1 M phosphate buffers. The cathodes are highlighted on the graphs. (**a**,**d**) Air-saturated solutions. (**b**,**e**) Deoxygenated conditions. The solutions were mechanically stirred at 300 rpm and the addition of H_2_O_2_ started at 250 s (C_H2O2_ = 2.2 × 0^−7^ M) with an interval of 50 s. Inserts show the SPES responses in the 2 × 10^−7^ M (at 250 s) to 3 × 10^−5^ M H_2_O_2_ concentration range. (**c**,**f**) Calibration curves under deoxygenated conditions (three independent experiments, error bars correspond to the standard deviations), GCE/FePc cathodes—black curves and symbols, and GCE/CGNP–FePc cathodes—red curves and symbols.

To evaluate the sensor ability of the new H_2_O_2_ SPES, the current responses of the cell composed of the Ni anode and the FePc- or GNP–FePc-modified cathode were tested, and the corresponding calibration curves to hydrogen peroxide at pH 3.0 and pH 7.4 were obtained. Moreover, the coiled Ni wire electrodes of different sizes were tested as anodes; however, the increase in the surface of the Ni anode did not influence the current output, and therefore, it was concluded that the cathode was the current limiting electrode of the SPES. The results are displayed in Figure 4.

As shown in Figure 4, the tests revealed that under aerobic conditions, the H_2_O_2_ SPES exhibited worse characteristics at a low concentration of H_2_O_2_ in both pH 3.0 and pH 7.4 buffers. This is explained by the fact that oxygen could have influenced the electrochemical properties of the cathodes and the anode, as discussed above.

Better results for the SPES were obtained under deoxygenated conditions (Figure 4b,e). Under deoxygenated conditions, cells with a FePc (or GNP–FePc) cathode reached a balanced condition faster than in aerobic solutions because oxygen was excluded. The sensors had better performance at pH 3 in comparison with pH 7.4, which is due to the decreased oxidizing capability of H_2_O_2_ at pH 7.4, different protonation conditions, and the redox behavior of FePc with a shift in the E_c_ to positive values at pH 3, as discussed above.

Table 2 summarizes the properties of the sensors. For both the FePc and GNP–FePc cells, the lowest detection limits (LDLs, found as 3.3 × S/b, where S is the standard deviation of the blank sample measurements and b is the slope of the calibration curve in a low concentration range) were found at pH 7.4 due to the smaller background current, but the highest detection limits (HDLs) were found at pH 3.0. At high concentrations of H_2_O_2_, the current decreased, which is likely due to the oxidative degradation of the FePc catalyst, Nafion [42,43], and electrode polarization. At pH 3.0, GNP–FePc shows a significantly better sensitivity and larger linear range towards H_2_O_2_ due to better conductivity, lower charge transfer resistance, better reversibility of the Fe(III/II)Pc reaction, and the favorable interface morphology with a better dispersion of the catalyst and a higher availability of the catalytic FePc sites.

Although it was previously reported earlier that the performance of the one-compartment H_2_O_2_ fuel cell composed of a FePc cathode catalyst and an Ni anode operated under basic conditions was significantly lower than that of the one-compartment H_2_O_2_ fuel cell operated under acidic conditions [29], the sensor ability of the H_2_O_2_ SPES was also evaluated under alkaline conditions at pH 12. The results are shown in Figure S8a. In addition to its low sensitivity, a significant drift of the sensor response was also observed.

Figure S8b illustrates that there was no response of the H_2_O_2_ SPES towards a number of common interfering substances. Figure S9a illustrates the stability of the sensor response and Figure S9b demonstrates that the SPES can be used repeatedly, although its performance decreases after about four days probably due to the operational instability of phthalocyanine. Moreover, it should be noted that iron phthalocyanine is light-sensitive, and thus, the sensor needs to be stored under dry and dark conditions.

### 3.6. Power Output Tests and Sensor Characteristics under the Control of External Variable Load Resistors

**Figure 5:**
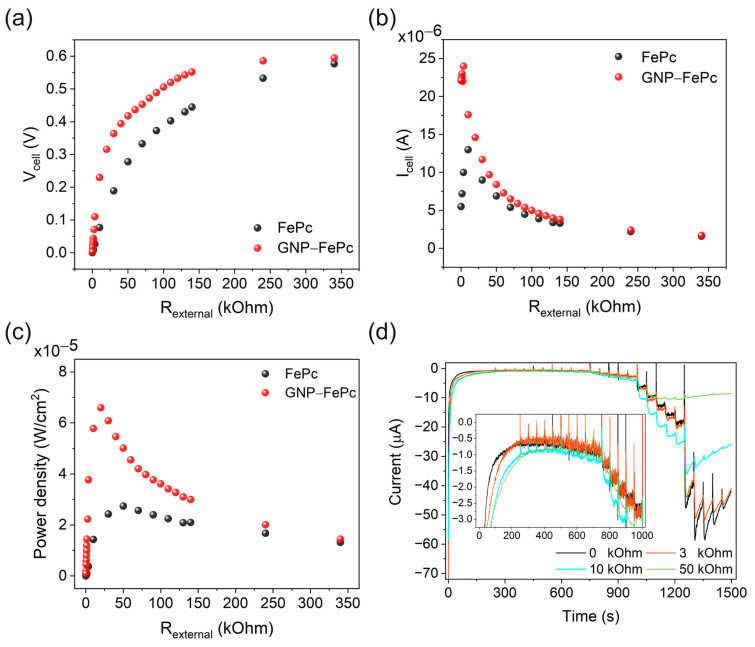
Representative curves showing the power and sensor performances with external load resistors: (**a**) voltage changes, (**b**) current changes, (**c**) power densities, (**a**–**c**) 3 mM H_2_O_2_, and (**d**) sensor performances with external loads 0, 3, 5, and 10 kOhm in deoxygenated buffer (pH 3). The solutions were mechanically stirred at 300 rpm and the addition of H_2_O_2_ started at 250 s (C_H2O2_ = 2.2 × 10^−7^ M) with an interval of 50 s to C_H2O2_ = 1.1 × 10^−2^ M. Inserts show a 2.2 × 10^−7^ to 3 × 10^−4^ M H_2_O_2_ concentration range.

Since SPES is based on a fuel cell, it was also important to test the performance with external loadings. The performance was thus examined in a deoxygenated pH 3.0 buffer with 3 mM H_2_O_2_. The results are shown in Figure 5.

Figure 5a shows the voltage changes at variable external load resistances. As can be seen, with the 340 kOhm resistor, cells with FePc and GNP–FePc catalysts show almost the same output voltage, which is near to the open circuit voltage. With a lower resistance, the cells switched to the discharge mode and the output voltage decreased, but the cell with the GNP–FePc catalyst showed a decreased voltage drop rate. Alongside the voltage changes, an increase in current was recorded (Figure 5b), and the cell with GNP–FePc shows a much higher peak current with a 3.4 kOhm external load resistor. As a combination result, the cell with the GNP–FePc catalyst shows the highest power density at 65.9 μW/cm^2^ with a 20 kOhm resistor (Figure 5c).

It is interesting that a stabilized output current was recorded with external resistance in the power tests. In order to study the influence from external loads on the sensor ability, tests with different resistors were therefore carried out and the results are shown in Figure 5d. It is worth noting that external resistance affected the current stabilization rate at the beginning (insert in Figure 5d). As discussed before, this current stabilization period at an initial state is due to the process on the double layer, the capacitive current, and changes on the Ni surface. It therefore takes more time for a cell with a high resistance to reach a balanced state due to the limited current flow. The cell with a load resistor of 3 kOhm showed a similar noise level to the cell without a resistor, but the noise level was significantly reduced with a 10 and 50 kOhm resistor. The sensor properties with external resistors are summarized in Table 3 and Figure S10.

As can be seen, with a low external resistance, the sensor property of the cells with the GNP–FePc catalyst did not change much, but with a 10 and 50 kOhm resistor, the linear detection range and the highest current of the cell were narrowed, which is because high resistance can hinder the electron transfer between an anode and a cathode. Nevertheless, the H_2_O_2_ SPES showed a much higher sensitivity with a 10 kOhm external resistor.

To explain this sensitivity and the noise level changes, the mechanisms of the cathode and the anode were considered. As discussed above, H_2_O_2_ can participate in different reactions on a cathode and an anode, involving different processes. As a result, different reaction rates on the anode and the cathode could have led to a disequilibrium of electron transfer from H_2_O_2_ to Ni, Ni to FePc, and FePc to H_2_O_2_ and further caused the instability of the signal and the electrode itself. Therefore, external loads affect or regulate a circuit current flow and stabilize the signal. However, the influence of this resistance was twofold as the following: at a low concentration of H_2_O_2_ (less than 1 mM), external resistance helped to increase the sensitivity and stability of H_2_O_2_ SPES, while at a higher concentration of H_2_O_2_, external resistance limited the performance due to an insufficient electron transfer, which means the oxidized cathode could not be reduced. Therefore, these results suggest that to achieve the application of fuel cell-based SPES, compatible anode and cathode materials are required and the working parameters need to be optimized.

Although there is a growing number of publications dedicated to the use of hydrogen peroxide fuel cells, only a few articles have presented self-powered H_2_O_2_ sensors based on the fuel cell configuration and principle so far. The characteristics of the reported H_2_O_2_ SPES are compared in Table 4. It demonstrates that the novel H_2_O_2_ SPES in this work has superior characteristics of a high sensitivity coefficient, low detection limit, and wide concentration range operating in a one-compartment configuration. 

### 3.7. Self-Powered Detection of Hydrogen Peroxide in Blood Serum

**Figure 6:**
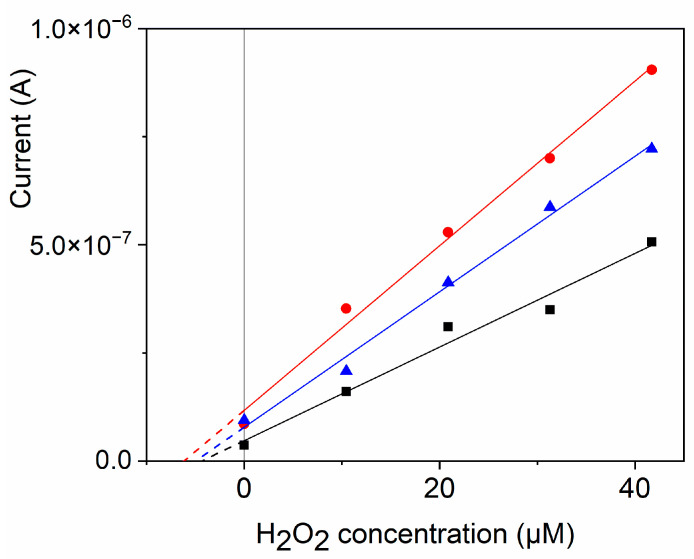
Example of the determination of the H_2_O_2_ concentration in blood serum (spiked with 5.2 µM H_2_O_2_) using SPES with the biomimetic cathode GCE/GNP–FePc by means of a four-point standard addition method, *n* = 3. Black solid line: linear fit, R^2^ = 0.97047; blue solid line: linear fit, R^2^ = 0.99051; red solid line: linear fit, R^2^ = 0.99091.

The practical applicability of the developed SPES for the analysis of complex real samples was tested by determining the H_2_O_2_ concentration in blood serum. The concentration of hydrogen peroxide in human blood was reported to vary from a low of 0.25 µM to a normal concentration range of 1–5 µM and a higher concentration range of 30–50 µM in inflammation or disease states [47]. Determination of exogenous H_2_O_2_ concentration by SPES was performed in heat-inactivated human blood serum diluted with 0.1 M PB (pH 3) 1:2 *v*/*v* using the standard addition method, as described in Section 2.3, because the matrix of the sample changed the analytical sensitivity. Earlier, it was found that commercial heat-inactivated serum samples did not contain a detectable amount of hydrogen peroxide due to the sample preparation and heat treatment [6]. Therefore, known amounts of hydrogen peroxide were added to the diluted serum samples and the concentration of hydrogen peroxide was determined using a four-point standard addition method. An example of this determination is presented in Figure 6 and the results are summarized in Table 5. The results demonstrate that the developed SPES is capable of determining the concentration of H_2_O_2_ in a complex biological matrix such as blood serum with high accuracy. Thus, this proof-of-concept study demonstrates a novel, simple, and efficient self-powered sensor system capable of detecting H_2_O_2_ in a complex matrix and highlights the perspective of this concept in the development of sensors.

## 4. Conclusions

In summary, a fuel cell-based H_2_O_2_ SPES using H_2_O_2_ as a single fuel was studied, and the morphology of cathode materials, electrochemical properties, and sensor ability were investigated. CV tests proved that the reduction currents for the electrodes modified with FePc were caused by the electrocatalytic function of biomimetic FePc. OCP tests proved the feasibility of the H_2_O_2_ SPES. The mechanism and detection function of the sensor was presented and discussed. The best analytical characteristics of SPES were achieved with the GNP–FePc cathode material, with a significantly higher sensitivity and larger working concentration range towards H_2_O_2_ due to a favorable interface morphology with a better dispersion of the catalyst and a higher availability of the FePc catalytic sites, higher conductivity, lower charge transfer resistance, and an improved reversibility of the Fe(III/II)Pc redox reaction on the GNP-modulated interface. The sensor characteristics under the control of a set of external variable load resistances indicated that optimized parameters were required for better sensor performance. The practical applicability and the matrix effect were verified by determining the H_2_O_2_ concentration in human blood serum. Although the detailed mechanism discussion depicted complex electrochemical properties and showed an interference effect from oxygen in the low H_2_O_2_ concentration range, this study highlights the simplicity in H_2_O_2_ SPES assembly, the improvement in the cathode interface, and, more importantly, presents the implementation and functional studies of a novel H_2_O_2_ SPES.

## Figures and Tables

**Table 1:** Electrochemical properties of FePc and GNP–FePc in deoxygenated buffer solutions.

pH	Fe(III/II)Pc				GNP–Fe(III/II)Pc			
	E_c_, V	E_a_, V	ΔE, V	−I_pc_/I_pa_	E_c_, V	E_a_, V	ΔE, V	−I_pc_/I_pa_
3.0	0.233	0.354	0.121	0.644	0.241	0.301	0.06	0.709
7.4	0.188	0.255	0.067	0.549	0.175	0.207	0.032	0.724

**Table 2:** Electrochemical sensor properties in deoxygenated buffer solutions.

	LDL, μM		HDL, mM		Sensitivity, A/(M·cm^2^)	
pH	3.0	7.4	3.0	7.4	3.0	7.4
**FePc**	1.0 ± 0.11	0.2 ± 0.04	0.91 ± 0.09	0.3 ± 0.05	0.117 ± 0.005	0.176 ± 0.016
**GNP** **–** **FePc**	0.6 ± 0.07	0.2 ± 0.03	3.0 ± 0.48	0.3 ± 0.06	0.198 ± 0.006	0.197 ± 0.021

**Table 3:** Electrochemical sensor properties of the cells with a GNP–FePc catalyst with different external resistors in a deoxygenated pH 3.0 buffer.

Resistor	LDL, μM	HDL, mM	Sensitivity, A/(M·cm^2^)
0 kOhm	0.6 ± 0.07	3.0 ± 0.48	0.198 ± 0.006
3 kOhm	0.6 ± 0.08	3.0 ± 0.60	0.197 ± 0.008
10 kOhm	0.8 ± 0.09	1.0 ± 0.12	0.350 ± 0.011
50 kOhm	1.0 ± 0.09	0.5 ± 0.07	0.262 ± 0.010

**Table 4:** Characteristics of the H_2_O_2_ SPES based on a fuel cell configuration.

Cathode	Anode	Compartment	pH; External Load	Detection Limit, µM	Working Range, mM	Sensitivity	Ref.
CoMn_2_O_4_ NPs/graphite	Bioanode from MFC ^[a]^	2	pH 7; 300 Ohm	40.2	1–1000	0.0132 A M^−1^	[44]
Graphite	Bioanode from MFC ^[a]^	2	pH 7; 300 Ohm	34.6	1–2000	0.011 A M^−1^	[4]
PB/NiHCF ^[b]^	Ag/AgCl in 0.1 M KCl	1	pH 6	–	2·10^−4^–1	0.59 to 0.65 A M^−1^ cm^−2^	[27]
Au/PB	Pt	1 (IDE ^[c]^)	H_2_O	0.02	Up to 0.2	0.00352 A M^−1^ cm^−2^	[45]
PB nanotubes	Pt	2	pH 7	0.1	Up to 0.08	0.048 A M^−1^ cm^−2^	[46]
PB MWCNT	Ni	1	pH 1; 100	1440	5–50	0.0375 A M^−1^	[13]
GNP–FePc	Ni	1	pH 3	0.6	Up to 3	0.198 A M^−1^ cm^−2^	This work
GNP–FePc	Ni	1	pH 7.4	0.2	Up to 0.3	0.197 A M^−1^ cm^−2^	This work
GNP–FePc	Ni	1	pH 3; 10 kOhm	0.8	Up to 1	0.350 A M^−1^ cm^−2^	This work

^[a]^ Microbial fuel cell; ^[b]^ Hexacyanoferrate; ^[c]^ Interdigitated electrodes.

**Table 5:** Determination of hydrogen peroxide in blood serum using H_2_O_2_ SPES.

H_2_O_2_ Added, µM	H_2_O_2_ Found (x ± Δx) ^[a]^, μM	R ^[b]^ (n = 3), %
5.2	5.2 ± 2.3	100.0
15.7	15.8 ± 8.0	100.6

^[a]^ The data are given as mean values and confidence limits about the mean, *n* = 3 and *p* = 0.95; ^[b]^ R—apparent recovery [34].

## Data Availability

The original contributions presented in this study are included in the article/Supplementary Materials. Further inquiries can be directed to the corresponding author.

## Supplementary Materials

The following supporting information can be downloaded at: https://www.mdpi.com/article/10.3390/bios14060290/s1, Section S1: Characterization Methods; Figure S1: Optimization of the GNP and FePc Ratio, EDX Analysis, and Elemental Mapping; Figure S2: Scan Rate Dependence; Figure S3: Cyclic Voltammograms of Bare GCE and GCE/GNP; Figure S4: Fitting Details of the EIS Tests; Table S1: Fitting Results of EIS tests of the Cathode; Section S2: The Properties of the Cathode under Aerobic Conditions; Figure S5: Voltammetric Characterization of the Cathode under Aerobic Conditions; Section S3: Electrochemical Studies of the Anode; Figure S6: Voltammetric Characterization of the Ni Anode under Deoxygenated Conditions; Figure S7: Voltammetric Characterization of the Ni Anode under Aerobic Conditions; Figure S8: Sensor Performance at pH 12 and Interference Study; Figure S9: Stability of the SPES Response; Figure S10: Examples of Calibration Curves of the H_2_O_2_ SPES with Different Resistors. References [48,49,50,51,52,53,54].
