# Correction: Zhang et al. A Study on the Mechanism and Properties of a Self-Powered H_2_O_2_ Electrochemical Sensor Based on a Fuel Cell Configuration with FePc and Graphene Cathode Catalyst Materials. *Biosensors* 2024, *14*, 290

**DOI:** 10.3390/bios14090452

**Published:** 2024-09-23

**Authors:** Yunong Zhang, Andreas Offenhäusser, Yulia Mourzina

**Affiliations:** 1 Institute of Biological Information Processing—Bioelectronics (IBI-3), Forschungszentrum Jülich, 52425 Jülich, Germany; yun.zhang@fz-juelich.de (Y.Z.); a.offenhaeusser@fz-juelich.de (A.O.); 2 RWTH Aachen University, 52062 Aachen, Germany

## Additional Affiliation

In this published publication [1], there was an error regarding the affiliations for Yunong Zhang. In addition to affiliation 1, the updated affiliations should include the following:

1. Institute of Biological Information Processing—Bioelectronics (IBI-3), Forschungszentrum Jülich, 52425 Jülich, Germany
2. RWTH Aachen University, 52062 Aachen, Germany

The authors state that the scientific conclusions are unaffected. This correction was approved by the Academic Editor. The original publication has also been updated.
