# Supplementary material for: A Study on the Mechanism and Properties of a Self-Powered H2O2 Electrochemical Sensor Based on a Fuel Cell Configuration with FePc and Graphene Cathode Catalyst Materials
Source: Biosensors (Basel). 2024 Jun 4;14(6):290. doi: 10.3390/bios14060290 (PMC11202192; doi:10.3390/bios14060290)
Supplement: Supplementary file 1 [file biosensors-14-00290-s001.zip › biosensors-3024995-supplementary.pdf]

biosensors

Supporting information

A Study on the Mechanism and Properties of a Self-Powered H_2O_2 Electrochemical Sensor Based on a Fuel Cell Configuration with FePc and Graphene Cathode Catalyst Materials

Yunong Zhang, Andreas Offenhäusser and Yulia Mourzina *

Institute of Biological Information Processing—Bioelectronics (IBI-3), Forschungszentrum Jülich, 52425 Jülich, Germany; yun.zhang@fz-juelich.de (Y.Z.); a.offenhaeusser@fz-juelich.de (A.O.)
* Correspondence: y.mourzina@fz-juelich.de; Tel.: +49-(0)2461612364

Section S1. Characterization methods

Figures S1 – S4

Table S1

Section S2. The properties of the cathode under aerobic conditions

Figures S5

Section S3. Electrochemical studies of the anode

Figures S6 – S7

Figures S8 – S10

References

S1. Experimental

S1. Characterization methods

The structure and element distribution was characterized by means of scanning electron microscopy (SEM) using a Magellan XHR SEM system equipped with an energy-dispersive X-ray spectroscopy (EDX) detector (FEI, Hillsboro, OR, USA).

UV-vis spectra were recorded with a PerkinElmer Lambda 900 spectrophotometer (PerkinElmer, Waltham, MA, USA). 1 cm quartz cuvettes were used in the tests, and DMF solution was used as a reference. A fresh FePc sample was prepared by diluting 0.6 mg/mL FePc in DMF to 0.06 mg/mL. 0.6 mg/mL FePc and GNP–FePc with 3 mg/mL GNP + 0.6 mg/mL FePc were prepared in DMF and rotated for 7.5 h. A 7.5 h FePc sample was then prepared by diluting rotated 0.6 mg/mL FePc to 0.06 mg/mL. For the centrifuged samples, 7.5 h FePc and GNP–FePc were centrifuged at 5000 rpm in 2 mL tubes for 5 minutes. The upper liquid was then taken out and diluted by 10 times.

Figures

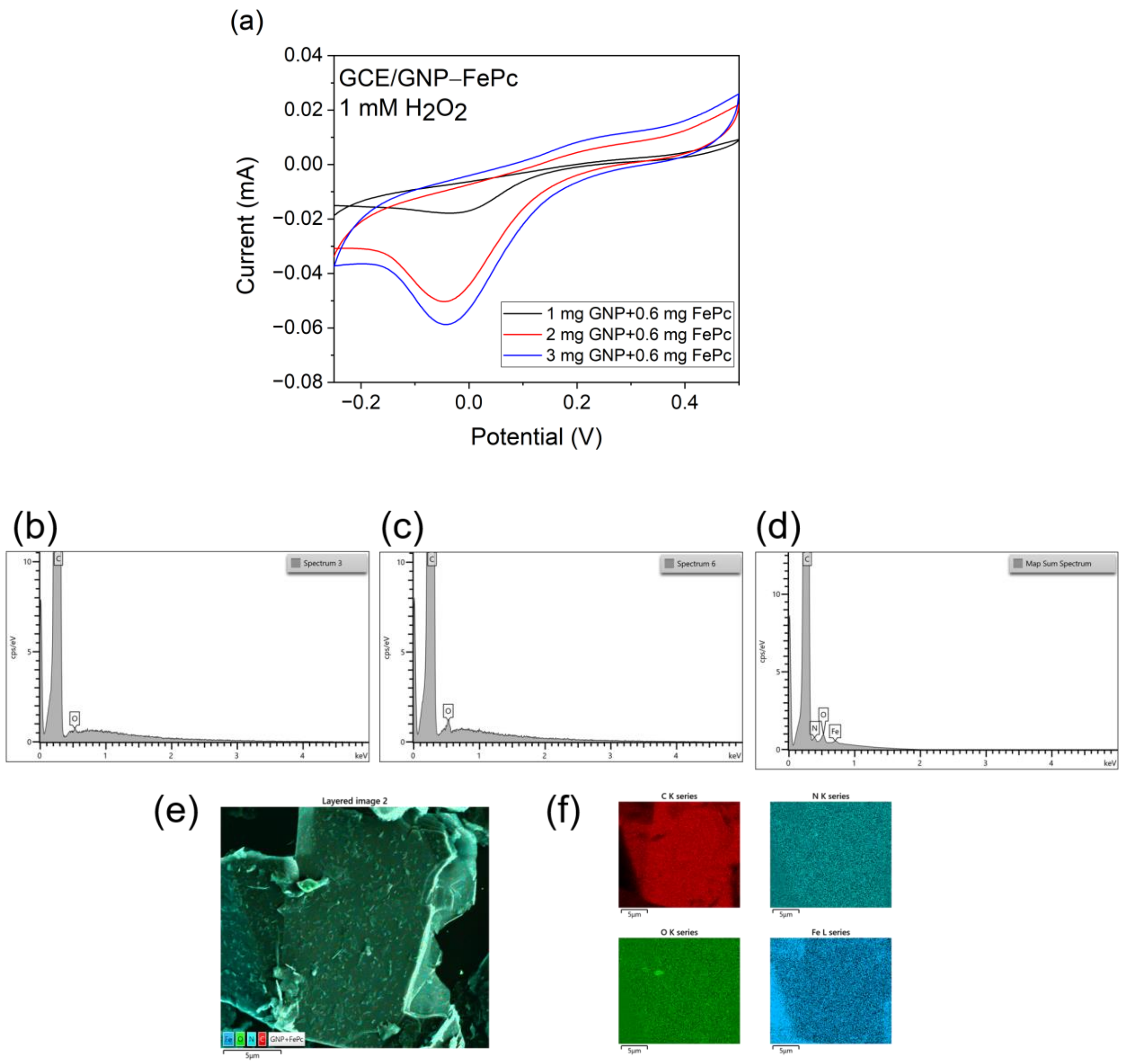

Figure S1. (a) Representative cyclic voltammogramms in 1 mM H_2O_2 with a 0.1 M phosphate buffer solution of pH 7.4 as a background electrolyte; scan rate 50 mV/s. Optimization of the GNP and FePc ratio based on the experiments on the reduction of hydrogen peroxide:.3 mg GNP–0.6 mg FePc was chosen. On the one hand, the increase in the H_2O_2 reduction current with the GNP(3 mg)–FePc composite compared to GNP(2 mg)–FePc composite was already less significant than compared to the GNP(1 mg)–FePc composite. On the other hand, larger amount of GNP or GNP–FePc were not employed, because Nafion could not completely cover the modified electrodes and the CVs became less reproducible.**(b)** – **(f)** EDX analysis of (b) GC; (c) GNP; (d) GNP–FePc; (e and f) elemental mapping images of GNP–FePc.

The EDX spectra of GC and GNP in Figure S1b and c show the signals of carbon and oxygen. Slightly higher signals of oxygen in the GNP originate from residual oxygen-containing functional groups on the edges of the platelets such as carboxyl, carbonyl, hydroxyl, and others. The EDX spectrum of the GNP–FePc composite on Figure S1d also displays a signal of Fe, which originates from the FePc in the composite material. Elemental mapping of the GNP–Fe in Figure S1e and f reveals a homogeneous distribution of the elements in the GNP–Fe composite material.

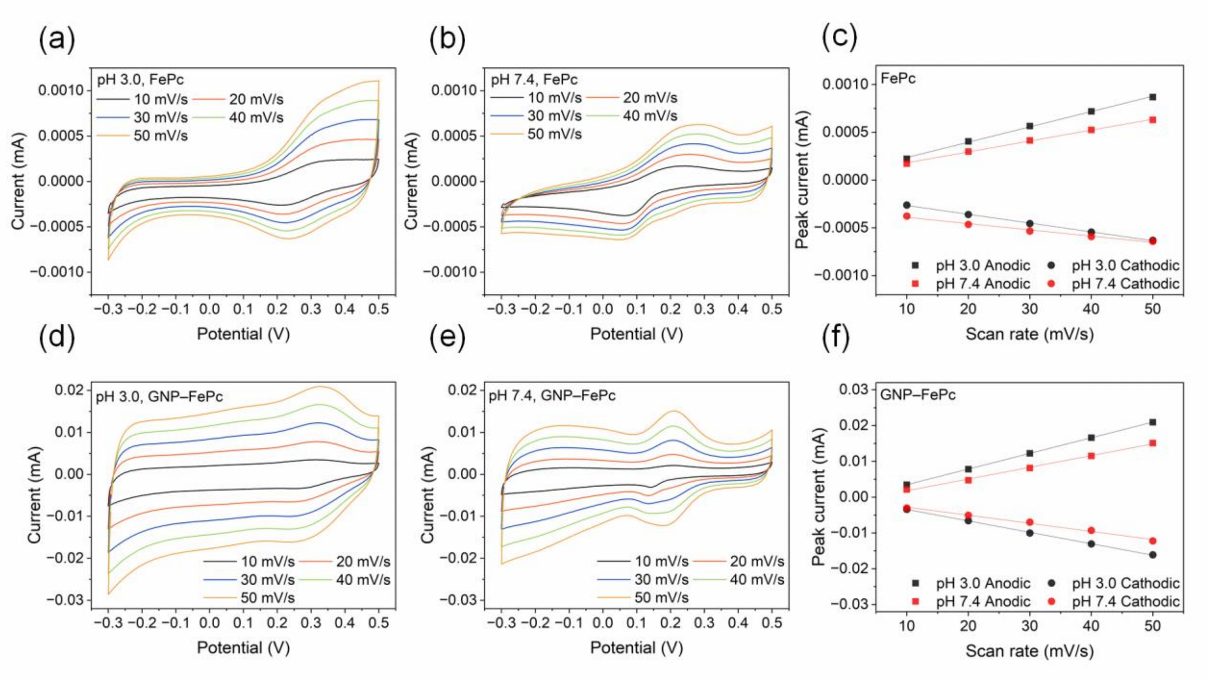

Figure S2. Reperesentative cyclic voltammogramms of GCE modified with FePc and GNP–FePc at different scan rates in deoxygenated buffers: **(a)** – **(c)** GCE/FePc at (a) pH 3 and (b) pH 7.4, (c) for pH 3 R^2_c=0.99889, R^2_a=0.99778 and for pH 7.4 R^2_c=0.98274, R^2_a=0.99825 ; **(d)** – **(e)** GCE/GNP–FePc at (d) pH 3 and (e) pH 7.4, (f) for pH 3 R^2_c= 0.99927, R^2_a= 0.99996, and for pH 7.4 R^2_c=0.98942, R^2_a=0.996.

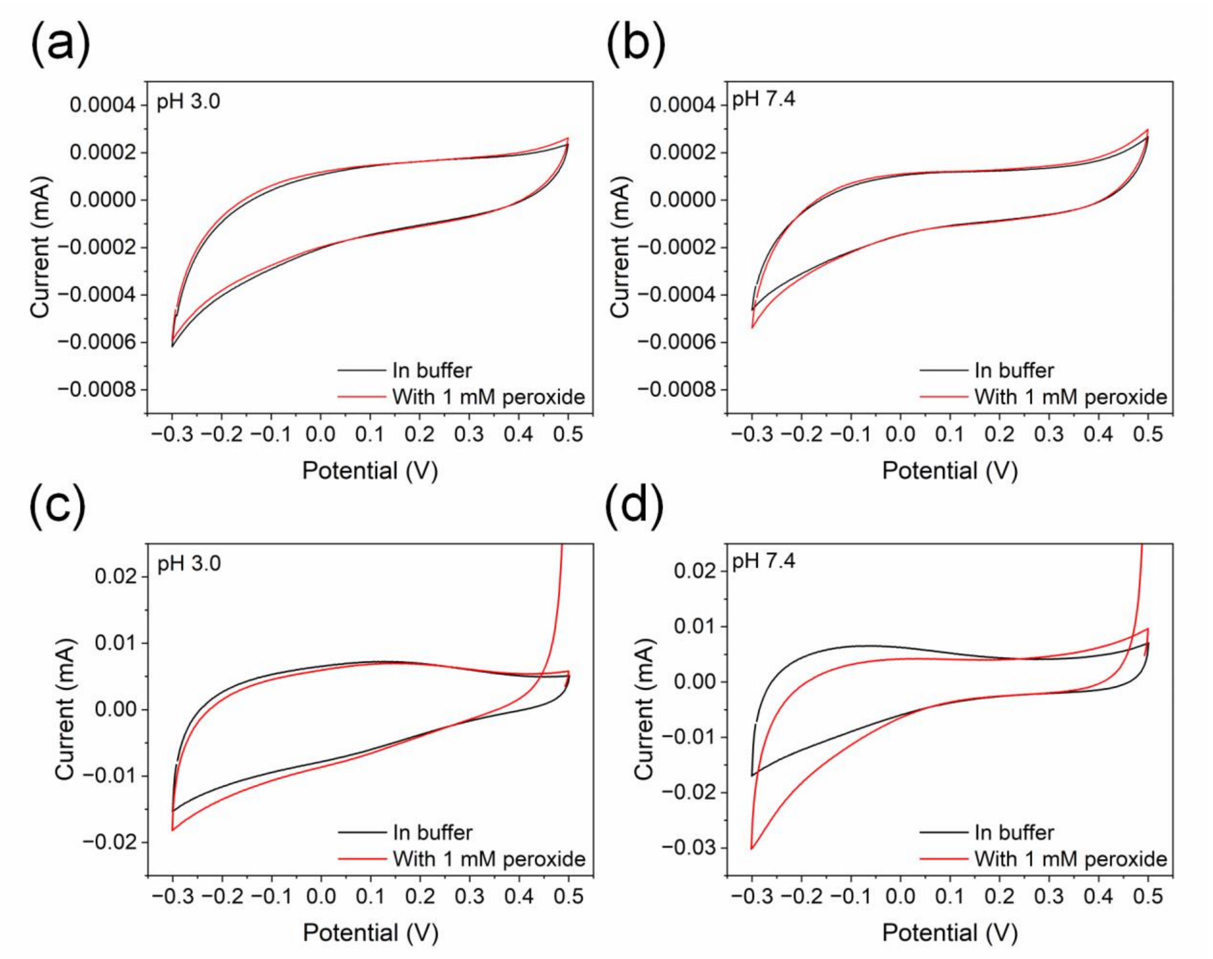

Figure S3. Cyclic voltammogramms of (a), (b) bare GCE and (c), (d) GNP–GCE in deoxygenated buffers. Scan rate 50 mV/s.

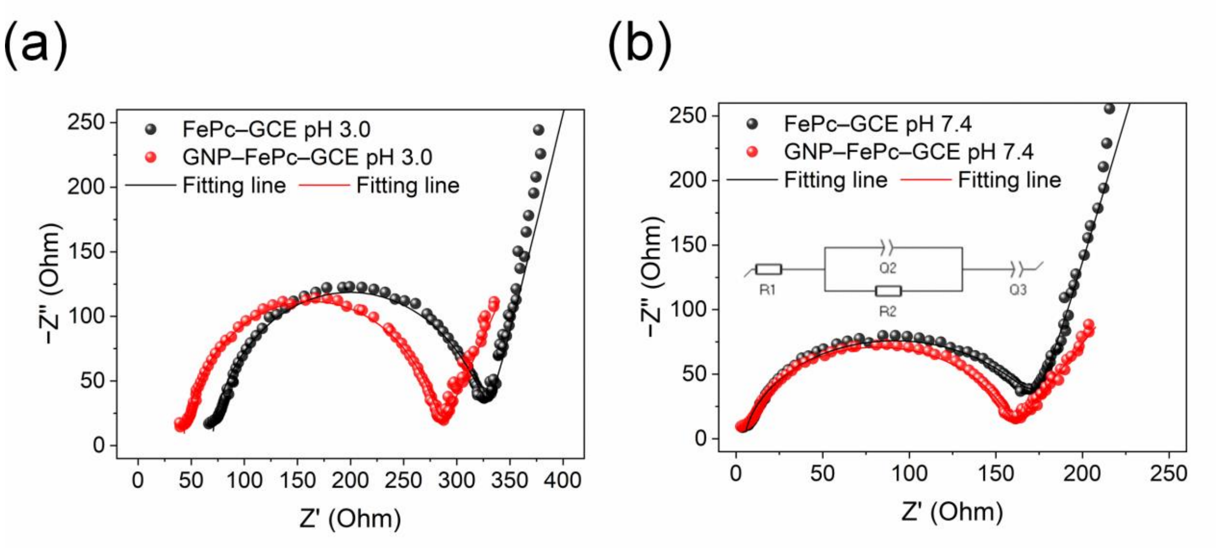

Figure S4. Fitting details of the representative EIS tests, the fitting model is displayed in (b).

Table S1. Fitting results of EIS tests of the cathode. [a]

Parameter	FePc		GNP–FePc	
	pH 3.0	pH 7.4	pH 3.0	pH 7.4
R1 (Ohm)	69.0	4.6	42.2	4.1
R2 (Ohm)	256.6	166.2	238.4	151.8
Q2 ($\times 10^{-9}$, $F{\cdot}s^{a-1}$); a2	18.7; 0.9396	40.6; 0.9192	18.4; 0.9511	29.3; 0.9459
Q3 ($\times 10^{-6}$, $F{\cdot}s^{a-1}$); a3	11.2; 0.8195	5.0; 0.8652	94.9; 0.6865	145.1; 0.6564

[a] For the EIS results, R1 is the solution resistance and R2 characterizes the charge-transfer resistance on the electrode. The results show that R1 and R2 are smaller for GNP–FePc than the cases with FePc, indicating a better conductivity with GNP modification and reversibility of the Fe(III/II)Pc reaction, as was also found in CV experiments (Table 1). Q2 and Q3 are the constant phase elements describing the double layers and belong to the electrode and Nafion layers.

Section S2. The properties of the cathode under aerobic conditions

As can be seen in Figure S5, oxygen has an influence on the electrochemical performance of both FePc– and GNP–FePc-coated GCEs in acidic and neutral buffers. In a pH 3.0 buffer, the reduction peak of oxygen can be found at 0.112 V for FePc, while it shifts to –0.0126 V at pH 7.4, which is reasonable, as the existence of H^+ promotes the oxygen reduction process. However, in the case of GNP–FePc, the reduction peak at pH 3.0 is not completed, but at pH 7.4, the peak is more pronounced. This change may indicate a more complicated mass and electron transfer, as GNP–FePc has a more complex interface. Moreover, oxygen can exist in the gaps between GNP pieces, leading to "internal oxygen", which can further influence electrochemical performance. Nevertheless, similar to the results obtained in deoxygenated buffers, the current of CV is much larger with GNP–FePc, indicating a larger electroactive area and more effective electron transfer between the cathode and the oxidant.

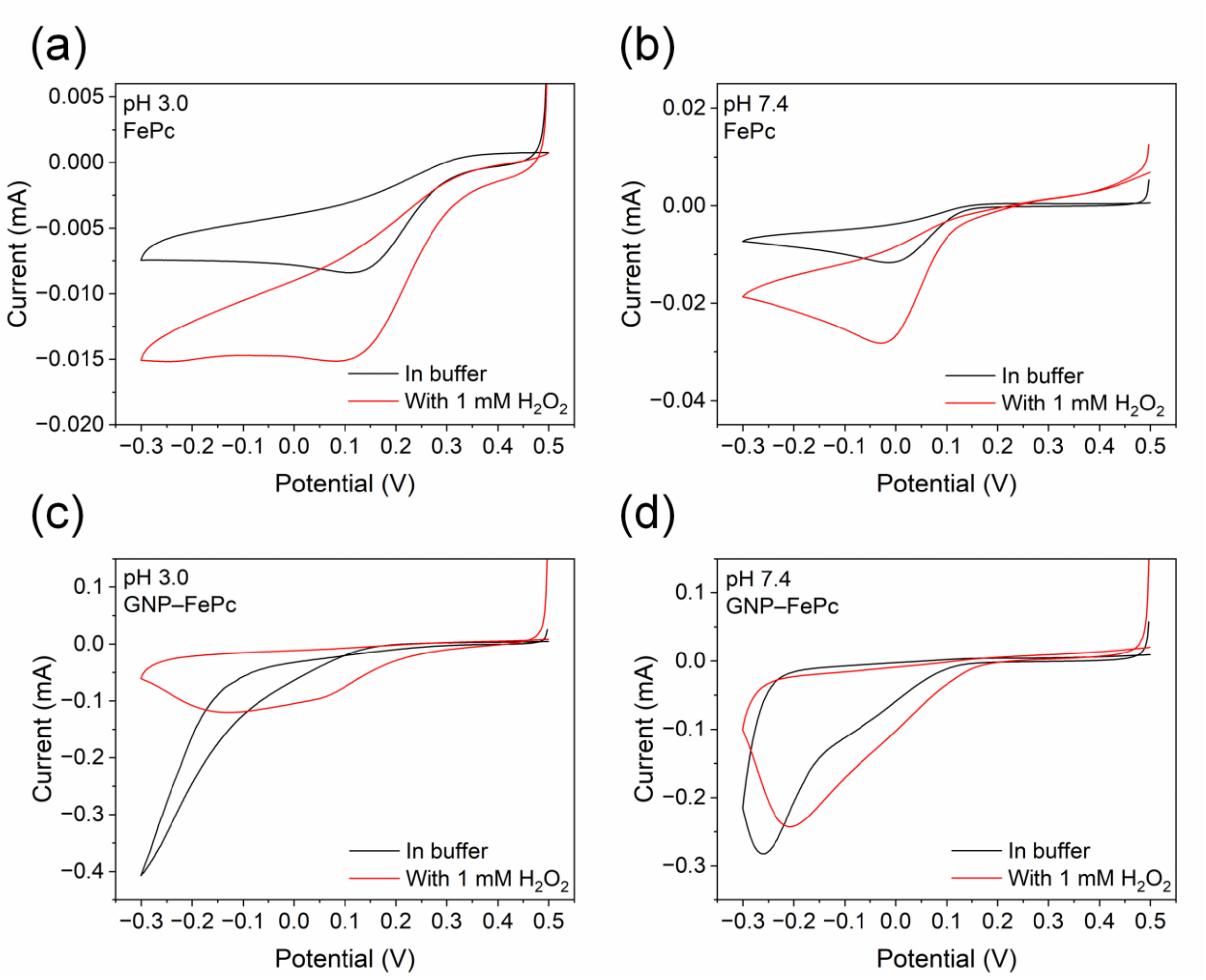

Figure S5. Voltammetric characterization of cathode under aerobic conditions. (a), (b) Representative CVs of FePc in different buffers and (c), (d) Representative CVs of GNP–FePc in different buffers without H_2O_2 (black lines) and with 1 mM H_2O_2 (red lines). Scan rate 50 mV/s. The results are discussed in Section S2.

Oxygen not only results in changes to the electrochemical performance of FePc and GNP–FePc in buffers, but to the CV results with 1 mM H_2O_2. As can be seen in Figure S5, with 1 mM H_2O_2, the peak currents increase in the cases with FePc in both conditions, which should be a combination of oxygen reduction and H_2O_2 reduction. However, in the cases with GNP–FePc, the peak currents become smaller. One possibility for this decrease is that the "internal oxygen" was consumed during the CV scan in buffers, which is more noticeable in the case of a high surface area of GNP. Thus, during the test without H_2O_2, the peak shows a combination of the reduction of "internal oxygen" and oxygen in buffers. After adding 1 mM H_2O_2, the peak shows a combination of the reduction of H_2O_2 and oxygen in buffers. Although the current changes in different ways, it is worth noting that the start potential shifts to a more positive range with H_2O_2 in all cases, which means that H_2O_2 can obtain electrons and be reduced on FePc and GNP–FePc more readily than oxygen. It is therefore possible to differentiate oxygen and H_2O_2 on FePc or GNP–FePc by applying different potentials. Similarly to the results obtained in deoxygenated buffers, the reduction process starts in the more positive range at pH 3.0, which is thermodynamically advantageous for the fuel cell construction.

Section S3. Electrochemical studies of the anode

For fuel-cell-based, self-powered electrochemical sensors, the electrochemical properties of the anode are also crucial, as H_2O_2 is oxidized on the anode and the electrons transfer from the anode to the cathode through external electrical connections. CV tests of

the anode in acidic and neutral solutions were therefore carried under anaerobic and aerobic conditions. The results are displayed in Figure S6 and Figure S7.

Figure S6 shows the CVs of Ni obtained in deoxygenated buffers. It worth noting that the CV tests reveal that Ni underwent different electrochemical oxidation processes in different buffers.

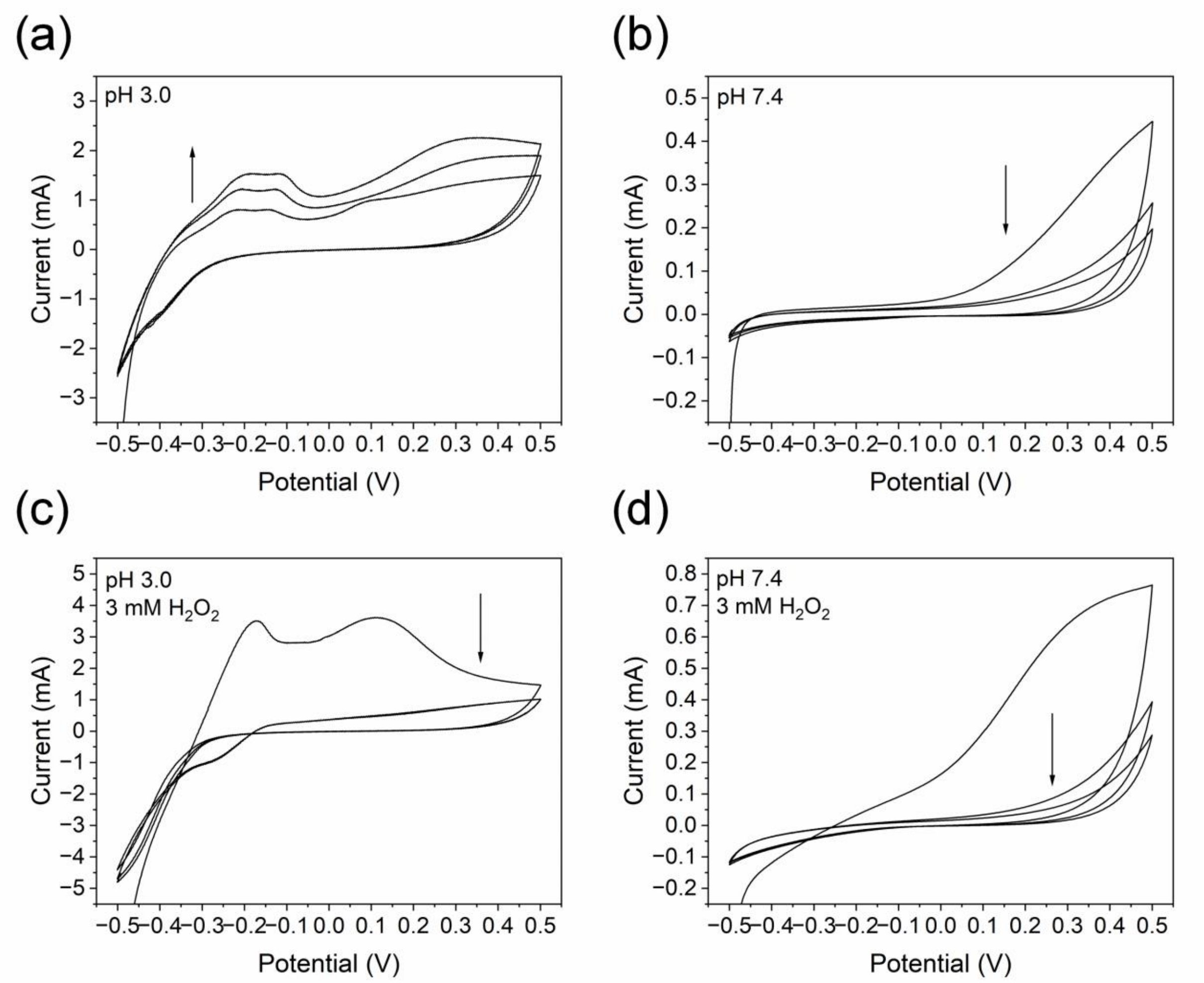

Figure S6. Voltammetric characterization of Ni anode under deoxygenated conditions. (a), (b) Representative CVs at pH 3.0 and 7.4. (c), (d) CV at pH 3.0 and 7.4 with 3 mM H_2O_2. Scan rate 50 mV/s.

As can be seen in Figure S6a, three anodic peaks appear at around –0.2 V, –0.1 V, and 0.3 V in a deoxygenated pH 3.0 buffer. While at more negative potentials, cathodic currents are observed due to the reduction of hydrogen and the oxidized Ni species.[1, 2] A complex reaction network to explain the Ni oxidation was proposed by Arvia.[2] The Ni anodic oxidation mechanism might be written as follows:[3]

$$Ni + H_2O \rightarrow (NiOH)_{ads} + H^+ + e^- \quad (S1)$$

$$(NiOH)_{ads} \rightarrow Ni(OH^+)_{ads} + e^- \quad (S2)$$

$$Ni(OH^+)_{ads} + H_2O \rightarrow Ni(OH)_2 + H^+ \quad (S3)$$

$$Ni(OH^+)_{ads, aq} + H^+ \rightarrow Ni^{2+} + H_2O \quad (S4)$$

$$Ni(OH)_2 + H^+ \rightarrow Ni^{2+} + H_2O + OH^- \quad (S5)$$

$$Ni(OH)_2 \rightarrow NiO + H_2O \quad (S6)$$

The appearance of the peak at –0.2 V might be caused by reaction (S1), where Ni was oxidized and the surface was covered by adsorbed $(NiOH)_{ads}$. Multiple anodic peaks in the region between –0.25 V and –0.05 V are due to a complex network of adsorption, oxidation, and desorption processes, with some examples presented by equations (S1) – (S6), as well as the oxidation of hydrogen adsorbed at cathodic potentials.[4] The anodic processes and the nature of the nickel oxide/hydroxide species depend on many factors and cannot be directly distinguished by CV experiments, an issue which remains the subject of discussion.[1, 2] The further oxidation of Ni and the formation of $Ni(OH)_2$ or NiO led to the appearance of the peak at 0.3 V.[2, 3, 5] Since the protons helped to refresh the Ni surface by chemical dissolution (reactions (S4) and (S5)) and removal of the Ni oxide/hydroxide species, an increased current intensity was obtained through multiple scanning cycles. However, the addition of H_2O_2, Figure S6c, led to the different oxidation behavior of Ni, as H_2O_2 can be reduced on the metal surface at a relatively negative potential according to the following reactions[5]:

$$H_2O_2 + 2e^- \rightarrow 2OH^- \quad (S7)$$

$$Ni + 2OH^- \rightarrow NiO + 2e^- + H_2O \quad (S8)$$

In addition to the fact that H_2O_2 participates in redox reactions at metal electrodes, decomposition of H_2O_2 by disproportionation is catalyzed at metal and metal oxide surfaces with the formation of surface-bound hydroxyl radicals and superoxide:[6]

$$H_2O_2 \rightarrow 2OH^\bullet \quad (S9)$$

$$OH^\bullet + H_2O_2 \rightarrow H_2O + HO_2^\bullet \ (pK_a(HO_2^\bullet)=4.88) \quad (S10)$$

$$2HO_2^\bullet \rightarrow O_2 + H_2O_2 \quad (S11)$$

As can be seen in Figure S6c, with 3 mM H_2O_2, an enhanced current intensity was recorded in the negative potential range (lower than –0.4 V), indicating a reduction of oxygen-containing species, which are present and formed in the system according to reactions (S7) – (S11), such as hydrogen peroxide, oxygen, and superoxide radical as well as Ni oxide and hydroxide. A stronger anodic peak subsequently occurred at –0.2 V, showing a more intensive oxidation process of reaction (S1), as the equilibrium of reaction (S1) was affected by OH^- from reaction (S7) and other oxygen-containing species. Superoxide radical anion from reactions (S9) – (S10) can also be oxidized on the electrode according to:

$$O_2^{\bullet -}(HO_2^\bullet) \rightarrow O_2 (+H^+) + e^- \quad (S12)$$

Moreover, a series of studies found that the oxidation of H_2O_2 proceeded via superoxide radical intermediate [7]. Unlike the results without H_2O_2, no peak appeared at –0.1 V, indicating that the formed $(NiOH)_{ads}$ was not removed from the Ni surface. A further anodic peak appeared at about 0.1 V, which is likely caused by the formation of Ni oxide/hydroxide. In the following scans, no anodic peak appeared at –0.2 V and 0.1 V, which means that a passive layer was formed on the Ni surface.[5] In the following scans, the oxidation of H_2O_2 took place on the passivated Ni surface with a start potential of about –0.15 V, which is more negative than the reduction of H_2O_2 on FePc and GNP–FePc catalysts. Consequently, $E_c - E_a > 0$, which enables the electrochemical cell to work in a power generation mode as a galvanic cell.

Unlike the CV behavior in a pH 3.0 buffer, Ni shows less pronounced changes in a pH 7.4 buffer. As shown in in Figure S6b, in the first scan cycle, CV reveals a strong oxidation process in the positive potential range. However, the current intensity decreased in the subsequent cycles, indicating a rapid passivation process on the Ni surface, which is due to the higher OH^- concentration in the pH 7.4 buffer in comparison with pH 3.0, and the failure of surface renewal by reactions (S4) and (S5). With 3 mM H_2O_2 (Figure S6d), the anodic current is larger than in the case without H_2O_2, which is due to the formation of OH^- and other oxygen species (reactions (S7) – (S11)), facilitating the

passivation of Ni and the formation of multilayer oxide films. Meanwhile, a low cathodic current appeared compared to the results in Figure S6b, which is due to the fact that H_2O_2 can be reduced in the negative potential range, as shown in reaction (S7). Moreover, the change in the redox ability of H_2O_2 also affects the cathodic intensity. Due to the lower oxidizing capacity of H_2O_2 at pH 7.4, lower reduction currents were obtained compared to the results at pH 3.0 (Figure S6c).

The electrochemical properties of the anode were also investigated under aerobic conditions and the results are displayed in Figure S7.

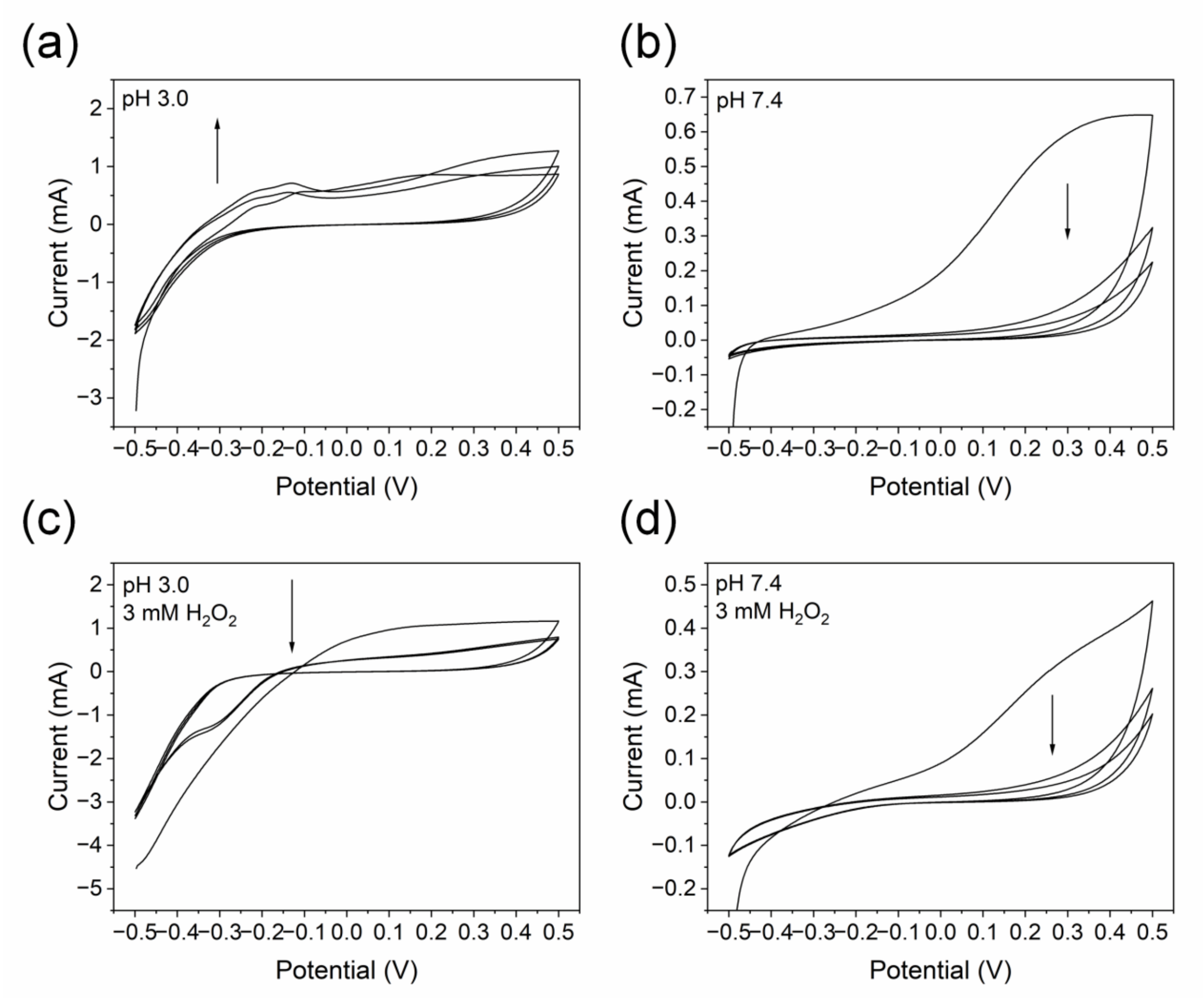

Figure S7. Voltammetric characterization of the Ni anode under aerobic conditions. (a), (b) Representative CVs at pH 3.0 and 7.4. (c), (d) CV at pH 3.0 and 7.4 with 3 mM H_2O_2. Scan rate 50 mV/s.

Similar oxidation peaks of Ni were obtained in CV tests under aerobic conditions. As shown in Figure S7a, CV exhibits a similar tendency but a reduced anodic current intensity. This can be attributed to the influence of oxygen on the passivation of Ni, which led to a more passive surface of the Ni anode. The reduced amount of Ni on the electrode surface thus participated in reaction (S1) and subsequent changes. However, it is worth noting that with 3 mM H_2O_2, no pronounced peaks occurred at –0.2 V and –0.1 V in Figure S7c, which means that oxygen facilitated the passivated surface formation of Ni with H_2O_2 by consuming protons in the oxygen reduction reaction and shifting the oxide/hydroxide equilibria at the electrode surface. The chemical condition on Ni surface is thus more alkaline, which could lead to the formation of $Ni(OH)_2$ and then further transform to NiO or NiO(OH).[3] In the following scans, the CV curves look almost identical to the results displayed in Figure S6c within the oxidation range of H_2O_2, which means that with the passivated Ni surface, oxygen has no significant influence on the electrochemical oxidation of H_2O_2 under the conditions of the experiment. Nevertheless, since oxygen can be

reduced together with H_2O_2, the reduction process of H_2O_2 on the passivated Ni surface can still be affected.

Figure S7b and d also show similar CV results to Figure S6b and d due to the advanced passivation of Ni at pH 7.4.

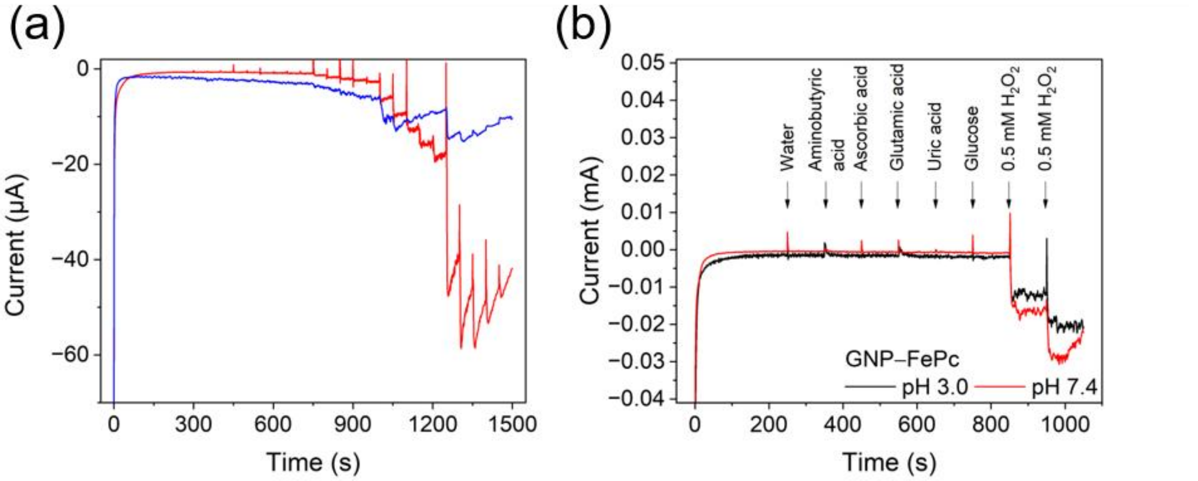

Figure S8. (a) Representative signal responses of the SPES with the GNP–FePc cathode at pH 3 (red line) and pH 12 (blue line) in deoxygenated conditions. The solutions were mechanically stirred at 300 rpm and the addition of H_2O_2 started at 250 s (C_{H2O2}=2.2×10^{-7} M) with an interval of 50 s.**(b)** Interference study of the H_2O_2 SPES with GNP–FePc in deoxygenated buffers (no external voltage was applied). The concentration of the chemicals after addition is 0.5 mM.

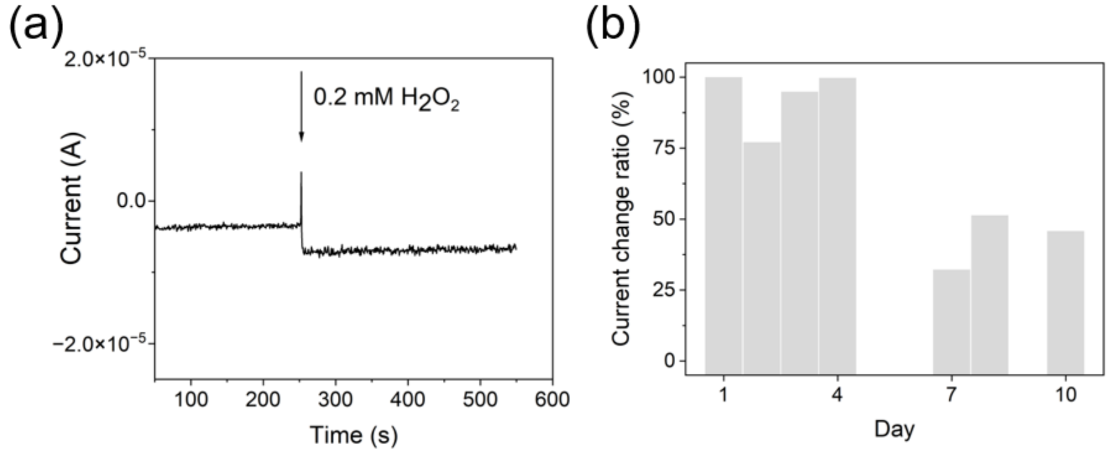

Figure S9. (a) Response of the H_2O_2 SPES to 0.2 mM H_2O_2. (b) Stability of the sensor response to 0.2 mM H_2O_2.

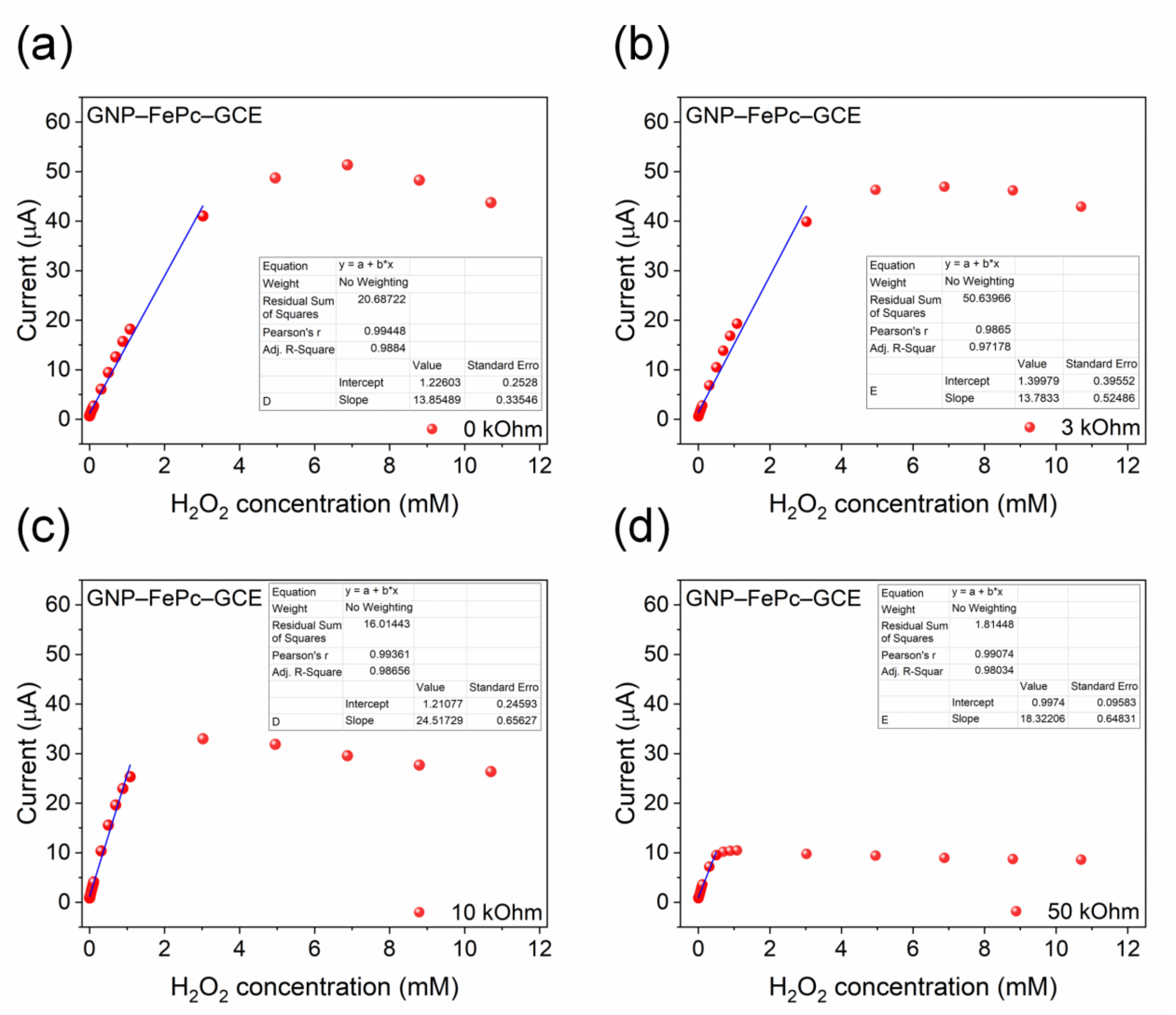

Figure S10. Example of calibration curves of the H_2O_2 SPES with different resistors (no external voltage was applied): (a) R=0 kOhm (R^2=0.9884), (b) R=3 kOhm (R^2=0.97178), (c) R=10 kOhm (R^2=0.98856), and (d) R=50 kOhm (R^2=0.98034).

References

1. Scherer, J.; Ocko, B.M.; Magnussen, O.M. Structure, dissolution, and passivation of Ni(111) electrodes in sulfuric acid solution: an in situ STM, X-ray scattering, and electrochemical study. Electrochim. Acta. 2003, 48, 1169-1191. doi: 10.1016/s0013-4686(02)00827-7.
2. Barbosa, M.R.; Real, S.G.; Vilche, J.R.; Arvia, A.J. Comparative potentiodynamic study of Nickel in still and stirred sulfuric acid-potassium sulfate solutions in the 0.4-5.7 pH range. J. Electrochem. Soc. 1988, 135, 1077.
3. Sanli, A.E.; Aytac, A. Electrochemistry of the nickel electrode as a cathode catalyst In the media of acidic peroxide for application of the peroxide fuel cell. ECS Trans. 2012, 42, 3-22.
4. Weininger, J.L.; Breiter, M.W. Hydrogen evolution and surface oxidation of nickel electrodes in alkaline solution. J. Electrochem. Soc. 1964, 111, 707.
5. Du, T.; Vijayakumar, A.; Sundaram, K.B.; Desai, V. Chemical mechanical polishing of nickel for applications in MEMS devices. Microelectron. Eng. 2004, 75, 234-241. doi: 10.1016/j.mee.2004.05.007.
6. Maier, A.C.; Iglebaek, E.H.; Jonsson, M. Confirming the formation of hydroxyl radicals in the catalytic decomposition of H2O2 on metal oxides using coumarin as a probe. ChemCatChem. 2019, 11, 5435-5438. doi: 10.1002/cctc.201901316.
7. Nairn, J.J., The electrochemical oxidation of hydrogen peroxide on nickel electrodes in phosphate buffer solutions. 2000, Massey University: Palmerston North, New Zealand.
